# Parallel spike trains analysis using positive definite kernels

**DOI:** 10.1186/1471-2202-15-S1-P29

**Published:** 2014-07-21

**Authors:** Taro Tezuka

**Affiliations:** 1 Faculty of Library, Information, and Media Science, University of Tsukuba, Tsukuba, 305-0821, Japan

## 

Since spikes are considered to be the fundamental element in neural systems, there has been a significant amount of work that analyzes the sequence of spike timings, or spike trains [1,2]. One important research question is to know how information is encoded in a spike train. There is an approach to construct a feature space that captures characteristics of spike trains, as is usually done in the field of pattern recognition. Another approach, which is increasingly becoming popular in recent years, is to find an appropriate similarity measure between spike trains [3,4]. This corresponds to constructing a model of information decoding done by the postsynaptic neurons.

One promising way to define such similarity measures is to use positive definite kernels [5-7]. Since positive definite kernels are generalizations of inner products, they enable application of various linear methods used in machine learning (including regression, classification, and dimension reduction) to spike trains. Positive definite kernels are extensively used in kernel methods.

While positive definite kernels on spike trains obtained from a single neuron have already been proposed [5-7], the extension of this to parallel spike trains obtained from multiple neurons has not been explored yet. Therefore, we defined such a kernel by extending the memoryless cross intensity kernel (mCI kernel) proposed by Paiva et al. [5]. To make the extension as natural as possible, we used a linear combination of cross-neuron interactions. We name the new kernel the linear combination of interactions kernel (LCIK) [9]. The parameters of the kernel can be set to make it positive definite.

We applied this kernel to publicly available *in vivo* recordings taken from the primary visual field in macaque monkey brains [8]. The LCIK performed better than other possible kernels defined on a set of spike trains. When the parameters of the kernel were estimated using spike trains obtained from real neurons, we obtained biologically plausible values. For example, the estimated time constant was near the value commonly used in neural modeling. This indicates the possibility of using such kernels to look for an appropriate model of neurons based on observed spike recordings.

We also simulated a simple neural network and generated spike trains to see the effect on the estimated values of the kernel parameters by changing the synaptic connection parameters. The result indicated that the kernel indirectly represents some of the internal parameters of the neural networks.
